# ^18^F-fluorothymidine PET for predicting survival in patients with resectable pancreatic cancer

**DOI:** 10.18632/oncotarget.24176

**Published:** 2018-01-12

**Authors:** Hinrich Wieder, Ambros J. Beer, Jens Siveke, Tibor Schuster, Andreas K. Buck, Ken Herrmann, Jens C. Stollfuss

**Affiliations:** ^1^ Department of Nuclear Medicine, Klinikum rechts der Isar, Technische Universität, München, Munich, Germany; ^2^ Centre for Radiology and Nuclear Medicine, Grevenbroich, Germany; ^3^ Division of Solid Tumor Translational Oncology, German Cancer Consortium, Partner Site Essen, Universitätsklinikum Essen, Essen, Germany; ^4^ Department of Family Medicine, McGill University, Montreal, Quebec, Canada; ^5^ Department of Nuclear Medicine, University of Würzburg, Würzburg, Germany; ^6^ Department of Nuclear Medicine, Universitätsklinikum Essen, Essen, Germany; ^7^ Institute for Diagnostic and Interventional Radiology, Klinikum rechts der Isar, Technische Universität München, Munich, Germany; ^8^ Department of Radiology and Nuclear Medicine, Klinikum Memmingen, Memmingen, Germany; ^9^ Department of Nuclear Medicine, Ulm University, Ulm, Germany

**Keywords:** FLT, PET, pancreas, predictive value, outcome prognosis

## Abstract

**Purpose:**

The aim of this study was to correlate preoperative 3'-deoxy-3'-[^18^F] fluorothymidine (FLT) uptake with the clinical outcome and survival in these patients after surgery.

**Materials and Methods:**

We performed a prospective analysis in 27 patients with adenocarcinoma of the pancreas (15 males, 12 females, mean age: 62 ± 13 years, range: 34 – 86 years). FLT PET (45 min p.i., 300 MBq FLT; ECAT HR+) images were acquired according to standard protocols. FLT uptake was quantified using standardised uptake values (SUV). Mean follow-up was 35 months (range 24-49). FLT uptake was correlated with survival using Martingale residual analysis.

**Results:**

Twenty-two patients died during follow-up. Mean overall survival was 18.8 months (SD: 12.7 months, 95% CI: 7.7, 26.5). FLT PET showed a mean SUV of 2.5 (range: 1.1 - 6.5). Martingale residual analysis revealed significant correlation between survival and FLT uptake (*p* = 0.045). The corresponding estimated hazard ratio per one-point increment of SUVmean was 1.298 (95% CI: 1.001, 1.685; *p* < 0.05).

**Conclusions:**

FLT PET allows risk stratification for death in patients with resectable pancreatic cancer prior to surgery.

## INTRODUCTION

Pancreatic cancer (PC) accounts for approximately 36,800 cancer deaths per year in the United States [1]. In industrialised countries, the incidence of adenocarcinoma of the pancreas ranks second after colorectal cancer among all gastrointestinal malignancies. The majority of patients present in the late stages of disease with locally advanced or metastatic tumours. Only 10–20% of these patients are candidates for resection with any potential for cure. Almost 50% of the patients have distant metastases at the time of presentation. Analysis of overall survival shows that the prognosis of PC is still extremely poor, despite the fact that 1-year survival has increased from 15% to 21.6% and 5-year survival has increased from 3% to 5% within the last decade [2–4].

Despite improvements in chemotherapy, surgical resection remains the only potentially curative treatment available for PC patients [1]. However, the outcome of patients undergoing surgery shows considerable variation [5–7]. The 5-year survival rate after radical resection is reported as between 10% and 29% and is difficult to predict using clinical TNM staging criteria [5–7]. Currently, there are no accurate clinical or morphological characteristics that can predict disease behaviour and prognosis in resectable PCs prior to surgery. Such markers would be useful guides for individual adjuvant therapy.

The thymidine analogue 3’-deoxy-3’-[^18^F] fluorothymidine (FLT), derived from the cytostatic drug azidovudine, has recently been developed for clinical positron emission tomography (PET) and allows *in vivo* imaging of proliferative activity in malignant tumours. The tracer has been shown to correlate with the Ki-67 labelling index in tumours *ex vivo* [8, 4]. Proliferative activity, in turn, is known to correlate with prognosis [9]. Seitz et al. showed the feasibility of FLT PET for imaging pancreatic cancer cell lines using an *in vitro* model [10]. Von Forstner et al. demonstrated FLT uptake in PancTuI and BxPC-3 pancreatic cancer cell lines [11]. FLT PET has also been shown to detect malignant pancreatic tumours and differentiate them from benign pseudotumours [12]. However, conclusions regarding the use of FLT in clinical studies remain controversial [12, 13]. To the best of our knowledge, no-one has yet investigated the value of preoperative FLT uptake to predict the outcome before surgical treatment is carried out. The aim of this study was, therefore, to correlate preoperative FLT uptake with the clinical outcome and survival of patients with PC after surgical treatment.

## RESULTS

### Histopathology

All 27 patients included in the final investigation had ductal adenocarcinoma of the pancreas. The tumour stage was pT1 in one patient, pT2 in 5 patients, pT3 in 18 patients, and pT4 in 3 patients. Twenty-one (78%) patients had positive nodes (pN1). None of the patients had evidence of distant metastases at the time of diagnosis (the cM0 status was clinically established on basis of CT and bone imaging). Eleven patients (41%) had positive resection margins (R1). The grading was G1 in 2 patients, G2 in 15 patients, and G3 in 10 patients.

### Follow-up and survival

Twenty-two patients died during follow-up. The mean overall survival was 18.8 months (SD: 12.7 months, 95% CI: 7.7, 26.5).

### FLT PET imaging

**Figure 1:**
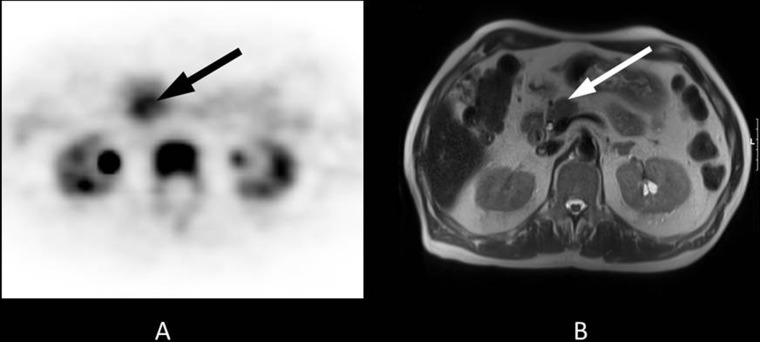
70-year-old patient with a 2-cm tumour in the pancreatic head Histopathology following Whipple procedure revealed a ductal adenocarcinoma of the pancreas. Final staging was pT3, pN0, cM0, R0. The patient received adjuvant treatment with gemcitabine 1000 mg/m^2^ according to a standard protocol (ESPAC 3) and had an ECOG Performance Status grade 1. The overall survival time was 9.3 months. (**A**) The axial PET image shows focal FLT uptake in the pancreatic head (black arrow, SUVmean 5.6). (**B**) The lesion is clearly visible on T2-weighted transverse MRI (white arrow).

**Figure 2:**
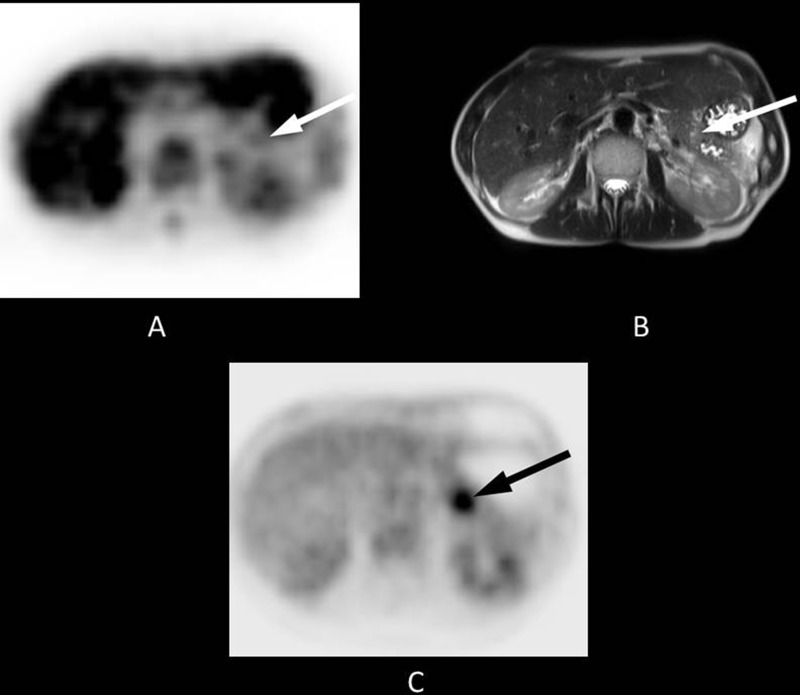
48-year-old patient with a 2-cm tumour in the pancreatic tail Histopathology following a distal pancreatectomy showed a ductal adenocarcinoma of the pancreas. Final staging was pT3, pN1, cM0, R1. The patient received adjuvant treatment with gemcitabine 1000 mg/m^2^ according to a standard protocol (ESPAC 3) and had an Eastern Co-operative Oncology Group (ECOG) Performance Status grade 1. The overall survival time was 17.1 months. (**A**) The axial PET image shows almost no FLT uptake in the area of the pancreatic tail (white arrow, SUVmean 1.4). (**B**) The lesion is clearly visible on T2-weighted transverse MRI (white arrow). (**C**) FLT uptake obviously does not correlate to that of ^18^F-fluorodeoxyglucose, as [18F]FDG PET shows substantial focal uptake in the area of the tumour (black arrow).

Twenty (74%) of the 27 FLT PET scans were rated as positive using visual analysis. Mean FLT uptake as measured by SUVmean was 2.4 (range 0.8–8.5). Corresponding maximum FLT uptake values ranged from 1.1 to 9.8, resulting in a mean SUVmax of 3.0. On the basis of the visual analysis, mean overall survival was 15.4 months (95% CI: 10.5, 19.5) Figure 1 in the PET-positive group and 20.9 months (95% CI: 14.1, 25.9) Figure 2 in the PET-negative group (*p* = 0.12, not significant). FLT PET scans did not show any additional lesions suggestive for metastatic disease. No lymph nodes could be separated from primary lesions. There was no significant difference in T-stage between PET positive and negative patients (80% of PET positive patients and 71% of PET negative patients had either a T3 or T4 lesion, n.s.).

### Correlation between initial FLT uptake and survival

**Figure 3:**
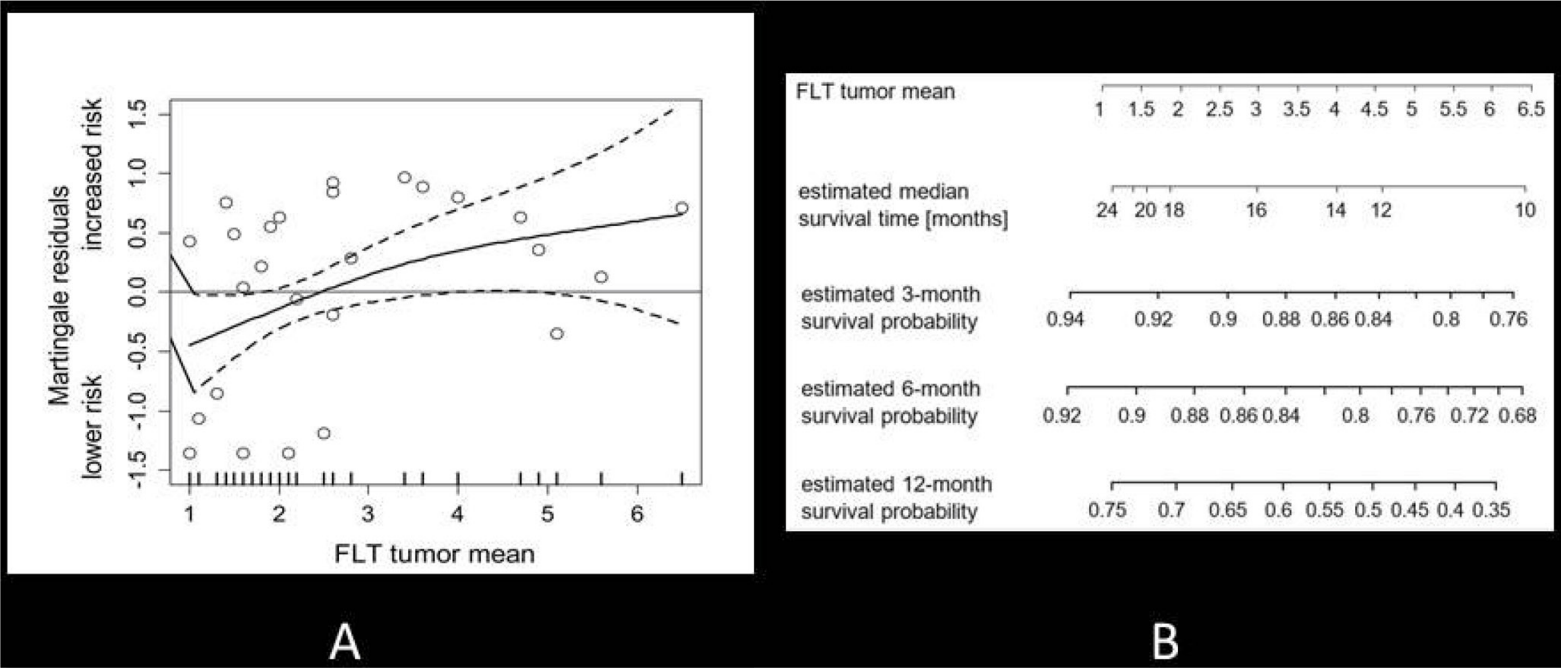
(**A**) Martingale residual analysis of FLT uptake (SUVmean) and the risk of death in all patients. Solid lines depict smoothing spline function with simultaneous 95% confidence bands. The corresponding estimated hazard ratio per one-point increment of FLT SUVmean was 1.298 (95% CI: 1.001, 1.3683; *p* < 0.05). (**B**) Mean tumour uptake is displayed in relation to median survival time. In addition, estimated survival probabilities are given for 3,6, and 12 months.

Figure 3A shows the approximate linear risk increments with increasing SUVmean of FLT uptake, using the Martingale residual analysis. The corresponding hazard ratio for FLT uptake as measured by SUVmean was 1.298 (95% CI: 1.001, 1.683; *p* < 0.05. There was a significant correlation between survival and initial FLT uptake. Figure 3B shows the corresponding estimated survival times in relation to tumour uptake as measured by the SUVmean.

## DISCUSSION

The thymidine analogue 3’-deoxy-3’-[^18^F] fluorothymidine has been shown to reflect proliferation-dependent retention of nucleosides in a number of different tumours, assessed non-invasively by PET [14–20]. However, the prognostic merit of pre-treatment FLT PET is still under investigation. To the best of our knowledge, this is the first study to evaluate the predictive value of FLT PET for overall survival in patients with resectable pancreatic adenocarcinomas. We have now demonstrated that increased preoperative FLT uptake is associated with a significantly increased risk of death in PC patients.

A recent report using FLT PET in patients with aggressive non-Hodgkin’s lymphomas showed that a high initial FLT uptake was a strong negative predictor of response to treatment and highly correlated to the International Prognostic Index [21]. However, initial FLT uptake failed to predict survival in that study, presumably due to the low number of deaths. In a recent investigation of 20 head-and-neck cancer patients, initial fluorothymidine and fluorodeoxyglucose (FDG) uptake correlated significantly with overall survival [22]. These findings are encouraging and warrant analysis in larger patient cohorts and in other tumour entities.

Only few data is available using preoperative FLT PET to evaluate the outcome in patients with pancreatic adenocarcinoma. In a recent study, Challapalli et al investigated the use of FLT PET/CT as an early response biomarker for gemcitabine-based chemotherapy in patients with advanced and metastatic pancreatic cancer. They concluded that FLT PET/CT might be useful to select a poor prognostic group who may benefit from novel therapeutic agents [23]. The data presented here should be regarded as indicative and further analysis needs to be undertaken before translating these results into clinical practice.

A number of important limitations have to be taken into account. Firstly, the case number is still relatively small. Secondly, the use of FLT is limited to few academic centres (at least in Europe). In addition, the sensitivity (and uptake) of FLT PET is lower than that of FDG PET and conventional non-invasive imaging procedures such as CT, MRI, and EUS [12, 24]. While adjuvant treatment after surgical resection of the tumours was standardized, there was heterogeneity with regard to palliative treatment administered during follow-up that might have an impact on overall survival. FLT PET has specific limitations in tissues with high physiological FLT uptake (e.g. bone marrow and liver) and is therefore of limited value in the detection of distant metastases [25]. Finally, FLT PET in our study was performed using a stand-alone PET scanner without simultaneous CT acquisition. Although CT or MRI scans were available to aid in ROI positioning, the use of hybrid PET/CT or PET/MRI systems may lead to greater accuracy. In particular, misinterpretation of intestinal uptake may give false positive FLT PET scans [26].

## MATERIALS AND METHODS

Thirty-five patients with malignant pancreatic tumours shown to be potentially resectable by conventional computed tomography (CT) or magnetic resonance imaging (MRI) were included in this prospective study between March 2007 and March 2009. Endoscopic ultrasound-guided (EUS-guided) fine needle aspiration and biopsy or brushing of the distal bile duct was performed if possible (*n* = 10). All patients underwent FLT PET within 30 days (mean 7 days) prior to surgical treatment. Seven patients were found have inoperable lesions at laparotomy and were excluded from further analysis. The remaining 29 patients underwent either a Whipple procedure (*n* = 20) or distal pancreatectomy (*n* = 9). Twenty-seven patients had ductal adenocarcinoma of the pancreas on histopathology. Two patients with intraductal mucinous lesions were excluded from the analysis. Thus, 27 patients were eligible for the final investigation and follow-up. All patients received adjuvant treatment with gemcitabine 1000 mg/m^2^ according to a standard protocol (ESPAC 3) and had an Eastern Co-operative Oncology Group (ECOG) Performance Status grade 0–2. All patients were in complete remission on first follow-up imaging after adjuvant therapy. The local ethics committee approved the study and all patients gave their informed consent. Table 1 shows the patient characteristics.

**Table 1:** Patient characteristics

Number of patients	27
Whipples Operation/Distal Pancreatectomy	100%
Adjuvant Chemotherapy	100%
Age [years]	
Median (range)	63 (34–86)
Interval: FLT–PET/Surgery [days]	
mean (range)	77 (6–200)

### PET imaging

3’-Deoxy-3’-[^18^F] fluorothymidine was synthesised as described previously [27]. Imaging was performed on a whole-body high-resolution PET scanner (ECAT HR+; Siemens/CTI; Knoxville, TN). The scanner simultaneously acquires 47 contiguous slices with a slice thickness of 3.4 mm. The in-plane resolution of transverse images was approximately 8 mm full width at half maximum (FWHM), with an axial resolution of approximately 5 mm FWHM. Static emission images were acquired 45 minutes after injection of approximately 300 MBq FLT (range: 270–340 MBq). Emission data were corrected for random coincidences, dead time and attenuation and were reconstructed by filtered back projection (Hanning filter with cut-off frequency of 0.4 cycles per bin). The matrix size was 128 x 128 pixels with a pixel size of 4.0 x 4.0 mm. The image pixel counts were calibrated to activity concentrations (Becquerel/gram [Bq/g]) and decay corrected using the time of tracer injection as reference.

### PET data analysis

All FLT PET scans were evaluated by two experienced nuclear medicine physicians/radiologists *(HW and JCS)* blinded to the clinical data and the results of other imaging studies. Discordant readings from the two observers were resolved by consensus. Circular regions of interest (ROIs) with a diameter of 1.5 cm were placed in the area with the highest tumour uptake as described previously [28]. In cases with low uptake, CT or MRI scans were used to place the ROIs using landmarks. Mean and maximum standardised uptake values (SUVmean and SUVmax) for all primary lesions were calculated from each ROI using the formula: SUV = measured activity concentration (Bq/g) x body weight (g)/injected activity (Bq). The Clinical Application Programming Package (CAPP; Siemens/CTI, Inc., Knoxville, TN) was used for defining ROIs and data analysis [29].

### Reference methods, clinical evaluation and follow-up

The histopathology served as the reference standard in all patients. Mean follow-up time was 35 months (range 24–49). Follow-up imaging was done using contrast enhanced CT chest-abdomen-pelvis, covering all regions except for the upper and lower extremities every 3 months.

### Statistical analysis

Statistical analyses were performed using PASW Statistics software (version 18.0; SPSS, Inc. Chicago, IL). Quantitative values were expressed as mean ± standard deviation and range. The Mann-Whitney *U* test was used to compare quantitative data between two independent samples. Spearman correlation coefficients were calculated to quantify bivariate correlations of measurement data. Exact two-sided 95 percent confidence intervals (CI) were reported for normally distributed estimates. Martingale residual analysis was performed to assess prognostic impact of continuous variables with regard to overall survival [30]. Smoothing spline equations have been fitted to the residual plots to depict the shape of the functional relationship between the continuous prognostic variable of FLT uptake and the risk of death. Based on the graphical assessment of the fitted residual curves, Cox proportional hazard models were fitted acknowledging possibly non-linear associations. Hazard ratio estimates were reported along with 95% confidence intervals. To illustrate differences between patients with low and high tracer uptake values, Kaplan Meier curves were plotted based on the sample median of the respective tracer uptake measurement (SUVmean or SUVmax). Kaplan-Meier curves depict 95% confidence bands for estimated survival probabilities and the *p*-value for the log-rank test for equal survival curves (Supplementary Figure 1). All analyses were two-sided tests and a *p*-value less than 0.05 was considered to indicate statistical significance.

## CONCLUSIONS

FLT PET allows risk stratification for death in patients with resectable PC prior to surgery. The data presented here justify further evaluation in larger cohorts to confirm preoperative FLT PET as a predictive tool in the management of patients with pancreatic cancer.

## SUPPLEMENTARY MATERIALS FIGURE


