# Supplementary material for: 18F-fluorothymidine PET for predicting survival in patients with resectable pancreatic cancer
Source: Oncotarget. 2018 Jan 12;9(11):10128–34. doi: 10.18632/oncotarget.24176 (PMC5839378; doi:10.18632/oncotarget.24176)
Supplement: Supplementary file 1 [file oncotarget-09-10128-s001.pdf]

^{18}F-fluorothymidine PET for predicting survival in patients with resectable pancreatic cancer

SUPPLEMENTARY MATERIALS

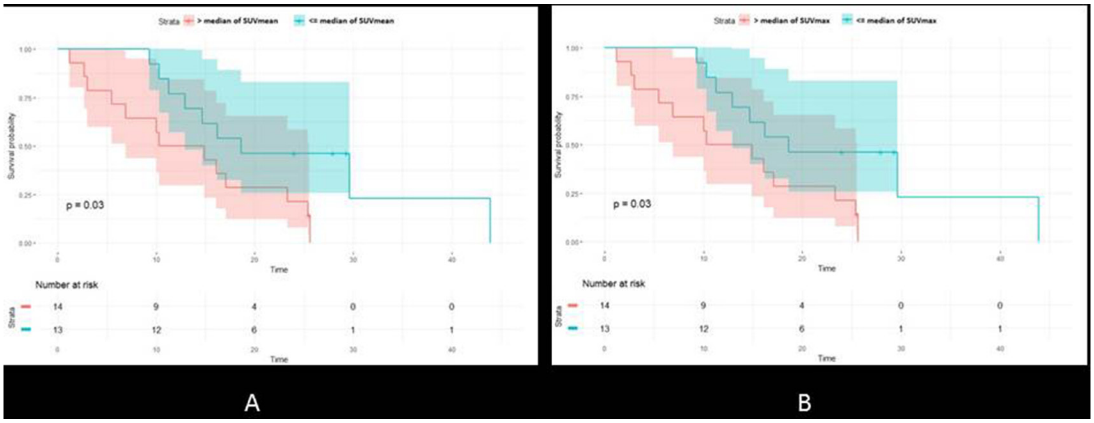

Supplementary Figure 1: Kaplan Meier curves based on the sample median of the respective tracer uptake measurement (**A**) SUVmean or (**B**) SUVmax) are displayed to illustrate differences between patients with low and high tracer uptake values. Ninety-five % confidence bands are given for estimated survival probabilities as well as the p-value for the log-rank test for equal survival curves.
